# Supplementary material for: Identification of Genes Associated with Gut Microbiota Metabolites in Type 2 Diabetes Mellitus Complicated by Acute Cerebral Infarction Based on Bioinformatics and Mendelian Randomization
Source: Biomedicines. 2026 Oct 17;14(10):2371. doi: 10.3390/biomedicines14102371 (PMC13702449; doi:10.3390/biomedicines14102371)
Supplement: Supplementary file 1 [file biomedicines-14-02371-s001.zip › Figure S1/genus.Alistipes.id.968 T2DM.pdf]

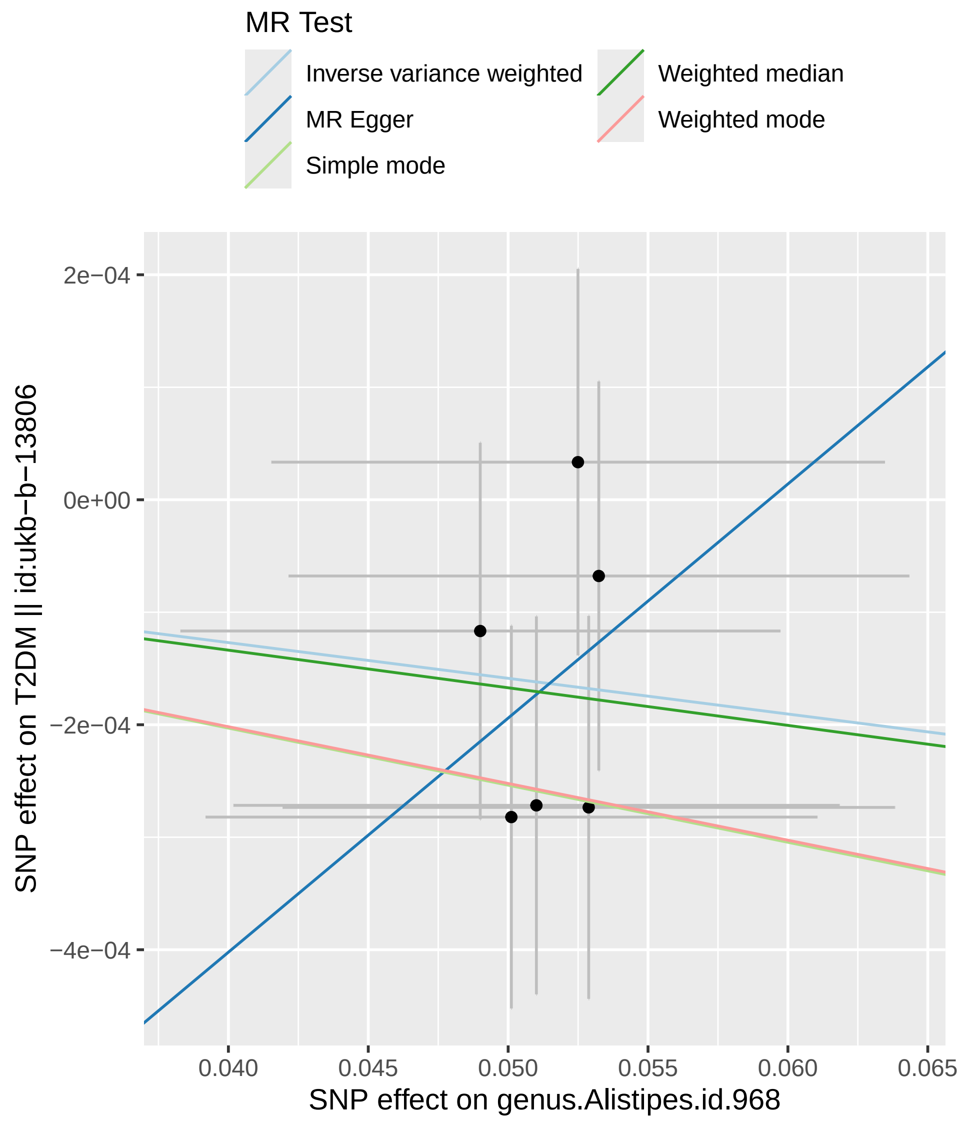

MR Test
Inverse variance weighted
MR Egger
Simple mode
Weighted median
Weighted mode
2e−04
0e+00
−2e−04
−4e−04
SNP effect on T2DM || id:ukb−b−13806
0.040
0.045
0.050
0.055
0.060
0.065
SNP effect on genus.Alistipes.id.968
